# Hydro-edaphic conditions defining richness and species composition in savanna areas of the northern Brazilian Amazonia

**DOI:** 10.3897/BDJ.5.e13829

**Published:** 2017-07-26

**Authors:** Maria Aparecida de Moura Araújo, Antônio Elielson Sousa da Rocha, Izildinha de Souza Miranda, Reinaldo Imbrozio Barbosa

**Affiliations:** 1 Universidade Federal de Roraima, Boa Vista - Roraima, Brazil; 2 Museu Paraense Emílio Goeldi, Belém - Pará, Brazil; 3 Universidade Federal Rural da Amazônia, Belém - Pará, Brazil; 4 National Institute for Research in Amazonia, Boa Vista - Roraima, Brazil

**Keywords:** Amazonian savanna, floristic survey, species richness, environmental factors, plant community

## Abstract

**Background:**

Studies on plant communities in the Amazon have reported that different hydro-edaphic conditions can affect the richness and the species composition of different ecosystems. However, this aspect is poorly known in the different savanna habitats. Understanding how populations and plant communities are distributed in these open vegetation areas is important to improve the knowledge about which environmental variables influence the occurrence and diversity of plants in this type of regional ecosystem. Thus, this study investigated the richness and composition of plant species in two savanna areas of the northern Brazilian Amazonia, using the coverage (%) of the different life forms observed under different hydro-edaphic conditions as a structural reference.

**New information:**

We report 128 plant species classified in 34 botanical families distributed in three savanna habitats with different levels of hydro-edaphic restrictions. In this study, the habitats are conceptually presented and they integrate environmental information (edaphic factors and drainage type), which determines differences between floristic composition, species richness and coverage (%) of plant life forms.

## Introduction

The Brazilian Legal Amazon presents non-forest formations (savannas/cerrados), covering an area of approximately 953.3 × 10^3^ km² (~19%) (Almeida et al. 2016), and they are considered regional ecosystems highly threatened due to large-scale human activities (Carvalho and Mustin 2017). The largest continuous area of these open vegetation formations in the northern of the region is located in Rio Branco – Rio Rupununi landscape complex, covering an area of 68,145 km² distributed between Brazil (42,706 km^2^), Guyana (14,500 km^2^) and Venezuela (10,939 km^2^) (Barbosa and Campos 2011). Most of these savanna areas are dominated by the herbaceous stratum (herbs and grasses), where trees and bushes may or may not be present (Beard 1953, Eden 1970). The Brazilian side of this large area of savanna covers the north-northeast of the state of Roraima, being locally known as "lavrado". This is a regional term widely used since the beginning of the 20th century, which defines the largest "enclave" of open areas in the Amazonian domain (Barbosa et al. 2007a, Nascimento and Carvalho 2016). The geological process of construction of this area in the state of Roraima is directly linked to tectonic events and to past fluctuations of climate occurred throughout the Quaternary. This process resulted in a “relict” landscape (remnant of old formations) which provide ecological patterns and biological diversity specific to this part of northern South America (Carneiro-Filho 1992).

Roraima’s savanna presents a mosaic of different phytophysiognomies with distinct structures and floristic compositions that vary from areas exclusively covered by herbaceous plants to areas with different concentrations of tree species (Miranda et al. 2002). Its phytophysiognomic diversity is very similar to that of the cerrado of Central Brazil (Barbosa and Fearnside 2005a, Miranda and Absy 2000), and some authors suggest that this whole set of open vegetation areas is determined by edaphic factors (Araújo and Barbosa 2007, Bueno et al. 2013, Vourlitis et al. 2013), which are associated with paleo and modern fires (Barbosa and Fearnside 2005b, Lopes et al. 2009, Pueyo et al. 2010, Moreira 2000).

In a broader and modern perspective, hydrological conditions (e.g. drainage type) have also been identified as important environmental factor, which determines the distribution of plant communities in the Amazon (Cordeiro et al. 2016, Higgins et al. 2011, Zuquim et al. 2014). However, for Roraima’s savanna, these studies are not totally conclusive, since the entire region is shaped by a mosaic of biogeomorphological systems that involve lacustrine formations with different hydro-edaphic conditions (Barbosa et al. 2007a, Carvalho et al. 2016). In these cases, environments with the same phytophysiognomic structure may have large variations in the richness and species composition, since hydro-edaphic variables can determine ecosystems with different vegetation structures and life forms. (Cavalcante et al. 2014).

In this context, the objective of this work is to make available data on the richness and species composition in Roraima’s savanna, using the plant coverage (%) as a proxy of their different life forms, and edaphic factors (fertility, texture) and soil drainage classes as predictor variables. Data were obtained from a floristic inventory carried out in 20 permanent plots distributed in two savanna areas in the state of Roraima (Monte Cristo and Água Boa), located in the northern of the Brazilian Amazon. The evaluation of the distribution of species and groups of species improves the understanding of the natural resources of these regional ecosystems and subsidizes intelligent ways to promote efficient public policies for the conservation of the Amazonian savannas.

## Project description

### Title

Ecology and management of the natural resources of Roraima´s Savanna

### Study area description

**Figure 1.**
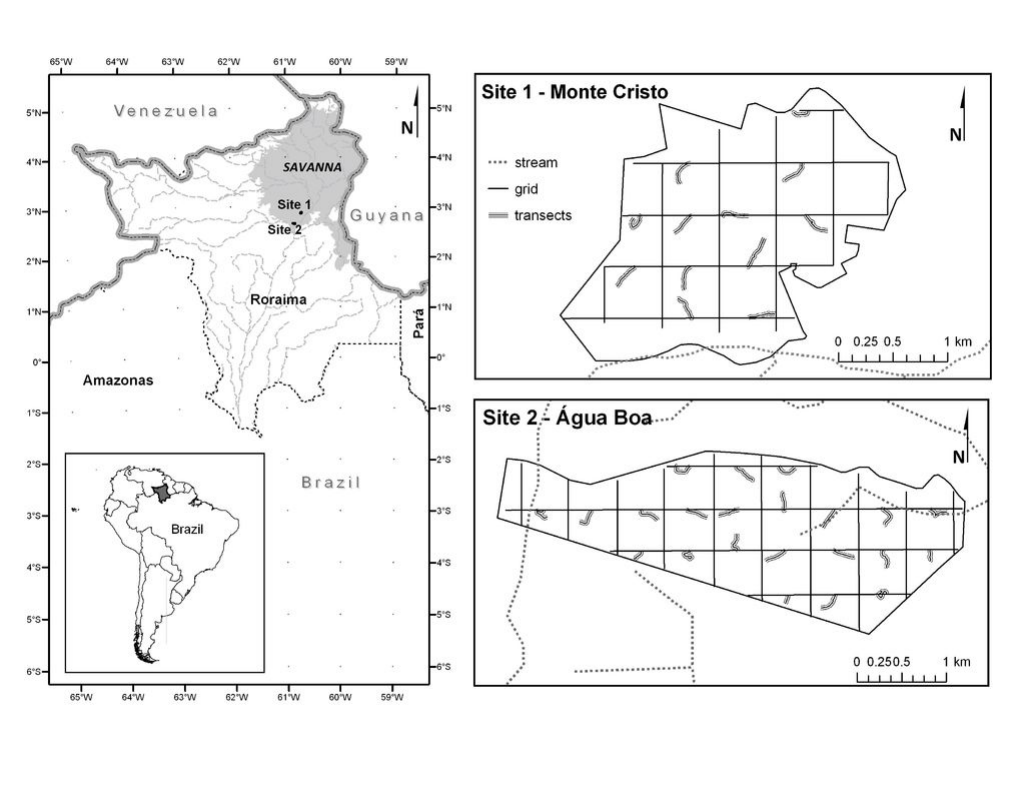
Location of the two PPBio modules studied in the Roraima’s savanna (*lavrado*), northern Brazilian Amazon.

The study was carried out in 20 permanent plots distributed in two research modules of the Program for Biodiversity Research (PPBio; https://ppbio.inpa.gov.br), managed by the Brazilian government, by means of the Ministry of Science, Technology and Innovation. Both modules are located in the municipality of Boa Vista, Roraima (Fig. 1):

**(i)** Campus do Cauamé, Monte Cristo region (MC): belongs to the Federal University of Roraima - UFRR (498 ha) and is located at ~15 km north of the city of Boa Vista (02°38’07”N to 02°40’11”N / 60°49’25”W to 60°52’28”W). It has 12 permanent plots (10 of which were randomly selected for this study). The relief of the study area is characterized as plan to wavy, due to its proximity to the Apoteri Formation (Benedetti et al. 2011). The vegetation of the area is defined as a mosaic of shrubby savanna (Campo Seco in Brazil) with savanna park-land (Savana Parque in Brazil), following the Brazilian vegetation classification system (Brazil-IBGE 2012).

**(ii)** Campo Experimental Água Boa (AB): belongs to the Brazilian Enterprise for Agricultural and Ranching Research - Embrapa Roraima (616 ha). It is located at ~36 km south of the city of Boa Vista (02°51’49”N to 02°53’06”N / 60°44’14”W to 60°42’27”W). It has 22 permanent plots (10 of which were randomly selected for this study). The vegetation of the area is typically a mosaic between shrubby savanna with wet grassland, interspersed with small patches of savanna park-land (Araújo and Barbosa 2007, Barbosa et al. 2007b).

Both modules are within the climatic type Aw, according to the Köppen classification, and present approximately the same average annual rainfall as that of the city of Boa Vista (~1,650 mm), with dry period defined between December to March, and the peak of the rainy season between May to August (Barbosa 1997).

### Funding

The study was supported by institutional project PPI-INPA (PRJ 015/122). MAMA was supported for a grant provided by CAPES, and RIB received CNPq productivity fellowship (Proc. 303081 / 2011-2). AESR and ISM were supported by the project "*Riqueza e diversidade de Poaceae e sua relação com variáveis ambientais em áreas de savanas da Amazônia*” (Museu Paraense Emílio Goeldi, MPEG - Belém/PA).

## Sampling methods

### Sampling description

**Figure 2.**
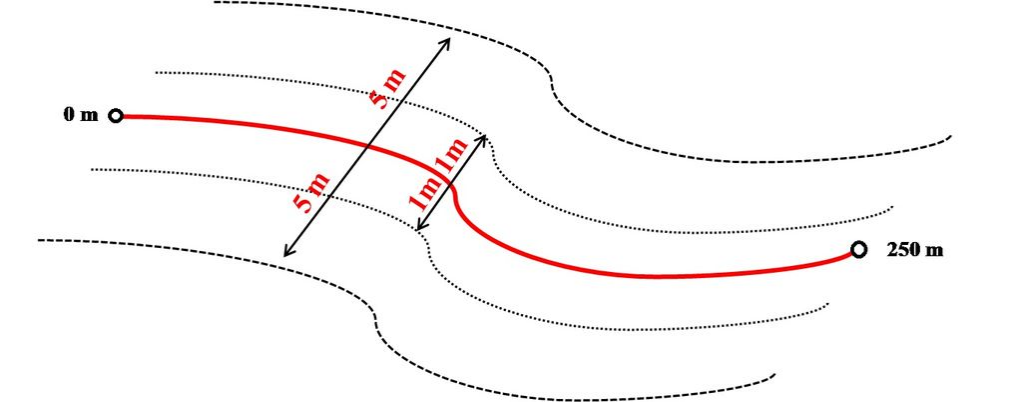
Schematic representation of the sample unit (permanent plot) for the sampling of herbaceous and sub-shrub (2 x 250 m) and tree and shrub (10 x 250 m) plants in the PPBio-Roraima’s savanna modules.

**(i) Plots Structure**: the 20 permanent plots are long (250 m in length), and they are oriented by the isoclines measured at the initial point of each of them (Fig. 2). This configuration is standard in PPBio and aims to minimize the variation in the abiotic factors that affect the different biological communities investigated in many studies (Magnusson et al. 2005, Magnusson et al. 2013, Pezzini et al. 2012). The floristic inventory was divided according to the life forms (herbaceous, sub-shrubs, shrubs and trees), which synthesizes the forms of life used by the international convention (Raunkiaer 1934) used in many other works in different areas of the Earth (Perrino et al. 2014, Silva and Batalha 2008). Herbaceous and sub-shrub plants were surveyed in a 2 m wide strip (1 m on each side of the plot central line), while tree and shrub plants were surveyed in a range of 10 m (5 m of each side of the plot central line) (Barbosa et al. 2010, Cavalcante et al. 2014).

**(ii) Floristic inventory**: floristic survey and collection of the botanical material were carried out between October 2012 to February 2013 in daily excursions between the end of the rainy season and the beginning of the dry season. All species were numbered and photographed. Excicates were prepared and deposited in the Herbarium of the Federal University of Roraima (UFRR - Boa Vista/RR). Unidentified specimens were subjected to the evaluation of specialists from the Herbaria of the Museu Paraense Emílio Goeldi (MPEG - Belém/PA), and from the Museu Integrado de Roraima (MIRR - Boa Vista/RR) for comparison with other materials.

**(iii) Plant coverage**: estimate of plants coverage was carried out by the Point Quadrat Method (Bullock 2006), according to the adaptations of Costa et al. (2005) and Magnusson et al. (2008). This method consisted by using a 2 mm thick and 1 meter height metal rod. The rod was vertically plotted in the soil along the transect line (250 m) that defines each plot. Each rod plotting was made at intervals of 50 cm throughout the permanent plot, totaling 500 points per plot.

**(iv) Hydro-edaphic variables**: fertility (sum of bases, Al, pH, P, Fe, Mn, Zn) and soil texture (% sand, % silt, % clay) data at 0-20 cm depth were obtained by previous samplings carried out in both modules by Pimentel and Baccaro (2011b) (UFRR / Cauamé) and Pimentel and Baccaro (2011a) (Embrapa / Água Boa). Each plot was classified according to the soil type (Gleysol, Oxisol, Ultisol) and to drainage class (well and poorly drained), following the Brazilian Soil Classification System (EMBRAPA-SOLOS 2006).

**(v) Data Analysis**: plots were clustered by the Ward Method using the coverage (%) of the plant species as a proxy of floristic similarity (correlation was used as similarity algorithm). Each group was defined as a specific phytophysiognomic type characterized by the soil type, drainage class, species composition, richness (S = number of species) and coverage (%) by life form. Plots were arranged using the multivariate NMDS technique (Non-metric Multidimensional Scaling) to identify the variables that better explain the species distribution and the organization of the phytophysiognomic structure. For this, the scores of Axis 1 (dependent variable) of the analysis were correlated with the environmental variables by simple linear regressions. All statistical analyses were performed using the R software (R Core Team 2016).

## Geographic coverage

### Description

This study was carried out in two PPBio savanna modules located in the municipality of Boa Vista, Roraima, to the north of the Brazilian Amazon: MC - Campus do Cauamé, Monte Cristo region (498 ha); 02°38’07”N to 02°40’11”N / 60°49’25”W to 60°52’28”W, and AB – Campo Experimental Água Boa (616 ha); 02°51’49”N to 02°53’06”N / 60°44’14”W to 60°42’27”W.

## Taxonomic coverage

### Description

**Description**: The scientific names of the species identified in the study were corrected by the search systems of the (i) Virtual Herbarium of the REFLORA Project (REFLORA – Brazilian Plants: Historic Rescue and Virtual Herbarium fo Knowledge and Conservation of the Brazilian Flora - http://reflora.jbrj.gov.br/reflora/PrincipalUC/PrincipalUC.do?lingua=pt), and (ii) The Plant List (http://www.theplantlist.org/). The families circumscription followed the APG-III (2009) classification.

We report 128 plant species (10,934 individuals) classified in 34 botanical families (Table 1). The species of higher richness were Cyperaceae (26 spp.; 20.3%), Poaceae (21; 16.4%), and Fabaceae (20; 15.6%). Only 11 species were identified to genus level only, and eight to family level. Of the total species observed, 61.7% (79 spp.) were herbaceous, 20.3% (26) were sub-shrubs, 8.6% (11) were shrubs, and 9.4% (12) were trees. Cluster analysis identified three distinct phytophysionomic clusters (habitats) according to the soil type and drainage class: (i) SAV-1, characterized by a mosaic of savanna park-land (Savana Parque in Brazil) with shrubby savanna (Campo Sujo in Brazil), prevailing well-drained red soil classes as Ultisol and Oxisol (n=7); (ii) SAV-2, shrubby savanna typically established in well-drained yellow Oxisol (n=8); (iii) SAV-3, wet grassland (Campo Limpo Úmido in Brazil) occurring in poorly drained soils (typically hydromorphic - Gleysol), where plots undergo seasonal flooding for 1 to 4 months every year (n=5). The highest species richness (S=90) was observed in SAV-1, followed by SAV-2 (71) and SAV-3 (61). Twenty-five species (generalists) occurred in the three groups, with special emphasis on the families Poaceae (*T. spicatus*, *P. carinatum*, *A. aureus*) and Cyperaceae (*R. barbata* and *B. capillaris*), both highly abundant and distributed in the three phytophysiognomic sets.

Coverage (%) with herbaceous plants (live + dead) was dominant in all the three groups (> 83%), with almost absolute predominance (~96%) in wet grasslands habitats (SAV-3) (Table 2). Coverage (%) of woody plants was higher in SAV-1 (2.5%) and SAV-2 (1.3%), where plots are well drained with no seasonal flooding problems. These two groups presented the highest concentration of exposed soil (13-14%), indicating lower densification among plants, despite they are richer and presenting higher tree and shrub coverage.

**Figure 3.**
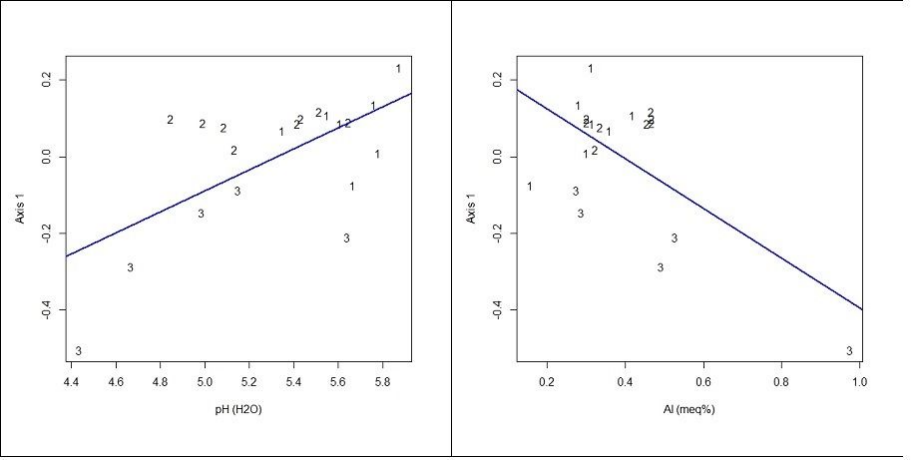
Linear regression indicating the correlation between the groups of habitats formed by floristic similarity of the plots (Axis 1 = scores NMDS 1) and the variables pH (H_2_O; Y = -1.4631 + 0.2747×X; R2 = 0.3591) and Aluminum (meq%; Y = 0.25472 - 0.65102×X; R2 = 0.3534). Groups of habitat: 1 – mosaic of savanna park-land with shrubby savanna / well drained (SAV-1); 2 – shrubby savanna / well drained (SAV-2); 3 – wet grassland / poorly drained (SAV-3).

Linear regression analysis indicated that pH (p < 0.003) and exchangeable Al (p < 0.003) are the edaphic variables that best explain the distribution of species within the three sets of plots (Fig. 3). In general, shrubby savanna (SAV-2) and mosaic of savanna park-land with shrubby savanna (SAV-1) are well drained habitats, with lower Al toxicity (0.306-0.391 meq%), lower acidity (pH = 5.3-5.6), and higher sum of bases (Ca+Mg+K = 0.25 to 0.43 cmolc kg). These characteristics indicate environments with lower hydro-edaphic restrictions associated with higher species richness. Conversely, wet grasslands (SAV-3) undergo seasonal flooding (poorly drained), have higher Al toxicity (~0.512 meq%), higher acidity (pH ~4.9), and lower sum of bases (~0.14 cmolc kg), resulting in environments with higher hydro-edaphic restrictions and lower species richness.

**Figure 4.**
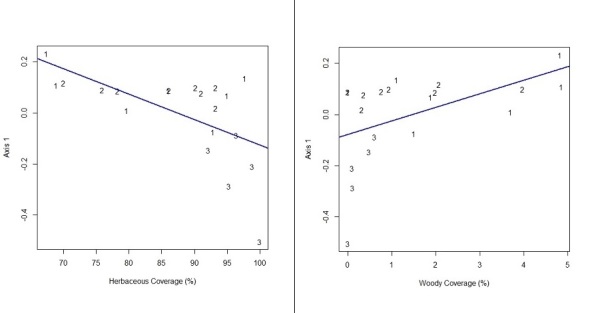
Linear regression indicating the correlation between the groups of habitats formed by the floristic similarity of the plots (Axis 1 = scores NMDS 1) and the coverage (%) of herbaceous plants (live + dead; Y = 0.873429 – 0.009996×X; R2= 0.3199) and woody plants (sub-shrub + shrub + tree; Y = -0.07886 + 0.05336×X; R2=0.2042). Groups of habitat: 1 – mosaic of savanna park-land with shrubby savanna / well drained (SAV-1); 2 – shrubby savanna / well drained (SAV-2); 3 – wet grassland / poorly drained (SAV-3).

Linear regression analysis also indicated that the phytophysionomical structure of habitats is partially explained by diversification of life forms and hydro-edaphic restriction. Predominance of herbaceous plants was significantly related (p < 0.005) to the habitats with lower richness and higher hydro-edaphic restriction (e.g., wet grassland) (Fig. 4). On the other hand, the coverage of woody plants (sub-shrub + shrub + tree) indicates to be related (p < 0.026) to habitats with greater diversification of life form and less hydro-edaphic restriction (shrubby savanna, and mosaic of savanna park-land with shrubby savanna).

Results suggest that the most restrictive savanna habitats (wet grasslands) are characterized by phytophysiognomies with less structural complexity in relation to the habitats conditioned by less restrictive hydro-edaphic factors (shrubby savanna, and mosaic of savanna park-land with shrubby savanna; both well drained). This effect directly influences the composition and life form of the species that inhabit the different habitats studied in this research (Table 3). In the plots of less hydro-edaphic restriction, the species *T. spicatus* (Poaceae) was predominant (24-54%), while *P. carinatum* (Poaceae) was predominant in wet grassaland (~18%). Similarly, the tree species with the highest coverage (%) were observed in the mosaic of savanna park-land with shrubby savanna (*C. americana* - Dilleniaceae, 2.7%) and in shrubby savanna on Yellow Oxisol (*B. crassifolia* - Malpighiaceae, 1.1%).). In the habitat formed by plots with seasonal flooding (wet grassland), only rare woody sub-shrubs and shrubs individuals were observed.

The results indicate that even with a small number of sample units (20) and study sites (2), Roraima’s savanna presents habitats with distinct floristic and structural characteristics, suggesting an ecosystem with high beta diversity associated with environmental heterogeneity. High beta-diversity was favored by the large number of species with low coverage (few individuals), which seems to be common in the Amazonian savanna (Magnusson et al. 2008). Although the habitats investigated in this study have common floristic elements, the hydro-edaphic factors determined the occurrence and the coverage of groups of species, providing different proportions between life forms in the different phytophysiognomic structures.

The present study highlights the environmental heterogeneity and the biological importance of Roraima’s savanna regarding the conservation of natural resources from the Amazon. In addition, it points out the need for greater investment in floristic inventories associated with greater diversification of sites, since this entire ecosystem has been rapidly modified by agribusiness (e.g. Aguiar et al. 2014). Further studies on Roraima’s “lavrado” are necessary in order to broaden the discussion about the demand for the creation of environmental protection areas as a public policy for the conservation of the largest savanna area in the Amazon (Pinto et al. 2008, Pinto et al. 2014).

## Usage rights

### Use license

Creative Commons Public Domain Waiver (CC-Zero)

### IP rights notes

These data can be freely used, provided their source is cited.

## Data resources

### Data package title

Hydro-edaphic conditions defining richness and species composition in savanna areas of the northern Brazilian Amazonia

### Resource link

http://ipt.sibbr.gov.br/sibbr/resource?r=savana_floristica

### Alternative identifiers

http://www.gbif.org/dataset/bf4641a7-a856-4061-8746-43a9e26db0cb

### Number of data sets

1

### Data set 1.

#### Data set name

Inventário florístico de 20 parcelas permanentes estabelecidas em áreas de savana do norte da Amazônia brasileira (Floristic inventory of 20 permanent plots localized on savanna areas of the northern Brazilian Amazonia)

#### Data format

Darwin Core Archive DwC-A

#### Number of columns

25

#### Description

Occurrences of plants with different life form identified during a floristic inventory in 20 permanent plots instaled in two savanna modules (Campo Experimental Água Boa and Campus do Cauamé), Boa Vista, Roraima, northern Amazonia. Dataset consist of the eml.xml, meta.xml and occurrence.txt containing the DwC-Attributes.

**Data set 1.** 

Column label	Column description
eventid	A identifier for the record (record code).
language	Language of the resource
institutionCode	Institution that has custody of the object or information about its registration.
occurrenceID	A identifier for the occurrence.
basisOfRecord	The specific nature of the data record.
collectionCode	The name or acronym of the collection or dataset from which the record is derived.
catalogNumbe	An identifier (preferably unique) for the record within the dataset or collection.
recordedBy	List of names of persons or organizations responsible for the registration of the original occurrence.
eventDate	The date or period during which an event occurred.
habitat	Description of the habitat in which the event occurred.
continent	The Continent of the occurrence.
country	The Country of the occurrence.
stateProvince	The State or Province of the occurrence.
county	The County of the occurrence.
locality	The location-specific description.
decimalLatitude	The geographical latitude in decimal degrees of the geographical center of a location.
decimalLongitude	The geographical longitude in decimal degrees of the geographical center of a location.
geodeticDatum	The ellipsoid, geodetic datum, or spatial reference system (SRS) in which the geographical coordinates given in decimalLatitude and decimalLongitude are based.
kingdom	Full scientific name of the kingdom in which the taxon is classified
family	Full scientific name of the family in which the taxon is classified
genus	Full scientific name of the genus in which the taxon is classified.
specificEpithet	Name of the species epithet of the scientificName.
scientificName	The full scientific name. It must be the name of lowest level taxonomic rank that was determined.
identificationQualifier	A brief phrase or standard term ("cf.", "aff.") to express the determiner's doubts about identification.
taxonRemarks	Comments or notes about the taxon or name.

## Figures and Tables

**Table 1.** List of species observed in both savanna modules of the PPBio/Roraima (AB = Campo Experimental Água Boa and MC = Campus do Cauamé, Monte Cristo), distributed in three categories of hydro-edaphic restrictions (SAV-1 = mosaic of savanna park-land with shrubby savanna in well-drained Ultisol and Oxisol; SAV-2, shrubby savanna in Yellow Oxisol; SAV-3, wet grassland in poorly drained soils, typically hydromorphic - Gleysol). Life form = Herbaceous, Sub-shrub, Shrub and Tree. The letter (x) indicates the presence of the species in the flooding categories. The signs (+) and (-) indicate that the species were present or absent, respectively, in the inventoried module. (*) indicates that the species occurred within the plot, but was outside the central transect line, and therefore was not considered in the coverage analysis.

**Family**	**Species**	**Habitat**			**Life Form**	**AB**	**MC**
		**Sav-1**	**Sav-2**	**Sav-3**			
Acanthaceae	*Ruellia geminiflora* Kunth	x		x	Herb	+	+
Anacardiaceae	*Tapirira guianensis* Aubl.*		x		Tree	+	-
Annonaceae	*Xylopia aromatica* (Lam.) Mart.*			x	Tree	+	-
Apocynaceae	*Himatanthus articulatus* (Vahl) Woodson.	x	x		Tree	+	+
Asteraceae	Asteraceae indeterminated	x	x	x	Herb	+	-
Connaraceae	*Connarus favosus* Planch	x	x		Shrub	+	+
	*Rourea grosourdyana* Baill	x	x		Shrub	+	+
Convolvulaceae	Convolvulaceae indeterminated	x			Herb	+	+
	*Evolvulus sericeus* Sw.	x	x		Herb	-	+
	*Ipomoea asarifolia* (Desr.) Roem. & Schult.	x			Herb	+	-
	*Merremia aturensis* (Kunth) Hallierf.	x	x		Herb	+	-
Cyperaceae	*Bulbostylis caespitosa* Peter	x	x	x	Herb	+	+
	*Bulbostylis capillaris* (L.) Kunthex C.B.Clarke.	x	x	x	Herb	+	+
	*Bulbostylis fasciculate* Uittien		x		Herb	+	-
	*Bulbostylis junciformis* (Kunth) C. B. Clarke	x	x	x	Herb	+	+
	*Bulbostylis lanata* (Kunth) Lindm			x	Herb	+	-
	*Bulbostylis oritrephes* (Ridl.) C. B. Clarke.			x	Herb	+	-
	*Bulbostylis paradoxa* (Spreng.) Lindm.	x	x	x	Herb	+	+
	*Bulbostylis truncata* (Nees) M. T. Strong.	x	x		Herb	+	+
	Cyperaceae indeterminated	x			Herb	+	+
	*Cyperus haspan* L.	x			Herb	-	+
	*Eleocharis filiculmis* Kunth.	x		x	Herb	+	-
	*Fimbristylis cymosa* R.Br.			x	Herb	-	+
	*Fimbristylis dichotoma* (L.) Vahl.	x			Herb	+	-
	*Lagenocarpus rigidus* (Kunth) Nees.			x	Herb	+	+
	*Rhynchospora barbata* (Vahl) Kunth.	x	x	x	Herb	+	+
	*Rhynchospora caespitosa* Huber.	x		x	Herb	-	+
	*Rhynchospora cephalotes* (L.) Vahl.	x	x		Herb	+	+
	*Rhynchospora filiformis* Vahl.	x		x	Herb	+	-
	*Rhynchospora globosa* (Kunth) Roem. & Schult.*			x	Herb	-	+
	*Rhynchospora nervosa* (Vahl) Boeckeler.	x			Herb	+	-
	*Rhynchospora riparia* (Nees) Boeckeler.		x	x	Herb	+	+
	*Scleria hirtella* Sw.	x	x	x	Herb	+	+
	*Scleria lagoensis* Boeckeler.	x	x	x	Herb	+	+
	*Scleria lithosperma* (L.) Sw.	x		x	Herb	+	-
	*Scleria reticularis* Michx.			x	Herb	+	+
	*Scleria rugosa* R. Br.	x		x	Herb	-	+
Dilleniaceae	*Curatella americana* L.	x	x		Tree	+	+
	*Davilla aspera* (Aubl.) Benoist		x		Subshrub	+	-
Droseraceae	*Drosera roraimae* (Klotzsch ex Diels) Maguire & J.R.Laundon.			x	Herb	+	-
Eriocaulaceae	*Syngonanthus gracilis* (Bong.) Ruhland			x	Herb	+	-
Erythroxylaceae	*Erythroxylum suberosum* A. St. Hil.		x		Tree	+	-
Fabaceae (Leguminosae)	*Aeschynomene histrix* Poir.	x	x		Subshrub	+	+
	*Aeschynomene paniculata* Vogel.	x	x		Subshrub	-	+
	*Bowdichia virgilioides* Kunth.	x	x		Tree	+	+
	*Chamaecrista diphylla* (L.) Greene.	x	x	x	Subshrub	+	+
	*Chamaecrista flexuosa* (L.) Greene.*	x	x		Subshrub	-	+
	*Chamaecrista hispidula* (Vahl) H. S. Irwin & Barneby.	x			Subshrub	-	+
	*Chamaecrista* sp.	x			Subshrub	-	+
	*Clitoria guianensis* (Aubl.) Benth.	x	x		Subshrub	+	+
	*Eriosema crinitum* (Kunth) G. Don.	x	x	x	Subshrub	+	+
	*Eriosema simplicifolium* (Kunth) G. Don.	x			Subshrub	-	+
	Fabaceae indeterminated		x	x	Subshrub	+	+
	*Galactia jussiaeana* Kunth.	x	x		Subshrub	+	+
	*Indigofera lespedezioides* Kunth.	x			Subshrub	+	+
	*Macroptilium gracile* (Benth.) Urb.*			x	Subshrub	+	+
	*Mimosa debilis* Willd.*	x			Subshrub	-	+
	Mimosa cf. pudica L.	x	x		Subshrub	-	+
	*Stylosanthes guianensis* (Aubl.) Sw.	x			Subshrub	-	+
	*Tephrosia* sp.	x			Subshrub	-	+
	Zornia (L.) Pers.	x	x		Subshrub	+	-
	*Zornia marajoara* Huber.*		x		Subshrub	+	+
Gentianaceae	*Coutoubea spicata* Aubl.		x		Herb	+	+
	Gentianaceae indeterminated			x	Herb	+	-
Haemodoraceae	*Schiekia orinocensis* (Kunth) Meisn			x	Herb	+	-
Lamiaceae	Lamiaceae indeterminated*	x			Herb	-	+
Lauraceae	*Cassytha filiformis* L.	x	x		Herb	-	+
Lentibulariaceae	*Utricularia adpressa* Salzm. ex A.St.-Hilaire & F.Girard			x	Herb	+	-
Loganiaceae	*Antonia ovata* Pohl	x	x		Tree	-	+
Lythraceae	*Cuphea antisyphilitica* Kunth.	x			Subshrub	-	+
	*Cuphea* sp.		x	x	Subshrub	+	-
Malpighiaceae	*Byrsonima coccolobifolia* Kunth.	x	x	x	Tree	+	+
	*Byrsonima crassifolia* (L.) Kunth.	x	x	x	Tree	+	+
	*Byrsonima* sp.*	x			Shrub	-	+
	*Byrsonima verbascifolia* (L.) DC.		x	x	Subshrub	+	+
Malvaceae	*Sterculia* sp.	x			Subshrub	-	+
	*Waltheria indica* L.	x			Herb	-	+
Melastomataceae	*Acisanthera crassipes* (Naudin) Wurdack.			x	Herb	+	-
	*Acisanthera hedyotoidea* Triana*			x	Herb	+	-
	*Acisanthera quadrata* Pers.	x			Herb	-	+
	*Miconia burchellii* Triana*		x		Shrub	+	-
	*Tibouchina aspera* Aubl.			x	Subshrub	+	-
	*Tibouchina gracilis* (Bonpl.) Cogn			x	Subshrub	+	-
Menispermaceae	*Cissampelos ovalifolia* DC.	x	x		Herb	+	+
Myrtaceae	*Eugenia punicifolia* (Kunth) DC.	x	x		Shrub	+	+
	*Myrcia* sp.	x			Shrub	-	+
	Myrtaceae indeterminated		x		Shrub	+	-
Ochnaceae	*Sauvagesia erecta* L.	x			Herb	-	+
Orobanchaceae	*Buchnera palustris* (Aubl.) Spreng.		x	x	Herb	+	-
	Orobanchaceae indeterminated	x	x		Herb	+	-
Poaceae	*Andropogon angustatus* (J.Presl) Steud.	x	x	x	Herb	+	+
	*Andropogon fastigiatus* Sw.	x	x		Herb	+	+
	*Andropogon selloanus* (Hack.) Hack.	x	x	x	Herb	+	+
	*Anthaenantia* sp.	x	x		Herb	+	+
	*Aristida torta* (Nees) Kunth.	x	x	x	Herb	+	+
	*Axonopus aureus* P.Beauv.	x	x	x	Herb	+	+
	Axonopus cf. purpusii (Mez) Chase.	x		x	Herb	+	+
	*Axonopus pubivaginatus* Henrard	x			Herb	-	+
	Elionurus cf. muticus (Spreng.) Kuntze	x	x	x	Herb	+	+
	*Elionurus* sp.	x			Herb	-	+
	*Mesosetum loliiforme* (Steud.) Hitchc.	x	x	x	Herb	+	+
	*Otachyrium succisum* (Swallen) Send. & Soderstr	x		x	Herb	+	+
	*Panicum arctum* Swallen	x	x	x	Herb	+	+
	*Panicum stenodes* Griseb.			x	Herb	+	-
	*Paspalum boscianum* Flüggé	x	x		Herb	+	+
	*Paspalum carinatum* Flüggé	x	x	x	Herb	+	+
	*Paspalum gardnerianum* Nees.		x		Herb	+	+
	*Paspalum hyalinum* Nees ex Trin.			x	Herb	+	-
	*Paspalum scrobiculatum* L.	x			Herb	-	+
	*Schyzachyrium sanguineum* (Retz.) Alston	x	x	x	Herb	+	+
	*Trachypogon spicatus* (L.f.) Kuntze.	x	x	x	Herb	+	+
Polygalaceae	*Polygala adenophora* DC.*		x		Herb	+	-
	*Polygala microspora* S. F. Blake.	x			Herb	-	+
	*Polygala subtilis* Kunth			x	Herb	+	+
	*Polygala trichosperma* L.	x		x	Herb	+	+
	*Polygala violacea* Aubl.	x	x	x	Herb	+	+
Proteaceae	*Roupala montana* Aubl.	x	x		Tree	-	+
Rubiaceae	*Genipa americana* L.*	x			Tree	-	+
	*Morinda tenuiflora* (Benth.) Steyerm		x		Shrub	-	+
	*Palicourea rigida* Kunth.	x	x		Shrub	+	+
	*Perama hirsuta* Aubl.*			x	Herb	+	-
	*Spermacoce capitata* Ruiz & Pav.	x	x		Herb	-	+
	*Spermacoce linearis* Willd. ex Roem. & Schult.	x		x	Herb	+	+
	*Spermacoce verticillata* L.	x	x		Herb	+	+
	*Spermacose* sp.		x		Herb	+	+
Salicaceae	*Casearia sylvestris* Sw.	x	x		Tree	-	+
Trigoniaceae	Trigonia villosa var. macrocarpa (Benth.) Lleras.	x	x	x	Shrub	+	+
Verbenaceae	*Lippia microphylla* Cham.	x	x	x	Shrub	+	+

**Table 2.** Coverage (%) of distinct plants by group of plots (habitat) and life form. Exposed soil represents a category where the point quadrat method did not detect any plant coverage, indicating the empty spaces in the environment. "n" represents the number of plots in each group.

**Group ofPermanent Plots**	**Bare Soil**	**Herbaceous**		**Subshrub**	**Shrub**	**Tree**	**Habitat**
		**live**	**dead**				
Sav-1 (n=7)	13.6±10.8	53.4±11.8	30.5±5.7	0.1±0.2	0.3±0.5	2.2±1.5	Mosaic Savanna Park-land withShrubby Savanna (Ultisol and Oxisol)
Sav-2 (n=8)	13.9±8.7	55.8±9.1	29.0±7.9	0.2±0.2	0.5±0.6	0.7±0.9	Shrubby Savanna (Yellow Oxisol)
Sav-3 (n=5)	3.3±2.9	77.0±5.0	19.5±3.2	0.3±0.3	0	0	Wet Grassland (Gleysol - Hidromorphic)

**Table 3.** Species with higher coverage (%) observed by life form and drainage class.

**Life Form**	**Sav-1**			**Sav-2**			**Sav-3**		
	Family	Species	Cover (%)	Family	Species	Cover (%)	Family	Species	Cover (%)
Herb	Poaceae	*Trachypogon spicatus*	23.77	Poaceae	*Trachypogon spicatus*	54.12	Poaceae	*Paspalum carinatum*	17.75
	Poaceae	*Mesosetum loliiforme*	14.03	Poaceae	*Paspalum carinatum*	9.39	Poaceae	*Paspalum hyalinum*	16.52
	Poaceae	*Axonopus aureus*	13.17	Poaceae	*Axonopus aureus*	8.58	Cyperaceae	*Rhynchospora barbata*	13.24
Sub-shrub	Lythraceae	*Cuphea antisyphilitica*	0.56	Fabaceae	*Aeschynomene histrix*	0.61	Malpighiaceae	*Byrsonima verbascifolia*	0.27
	Fabaceae	*Galactia jussiaeana*	0.18	Fabaceae	*Eriosema crinitum*	0.25	Rubiaceae	*Tibouchina aspera*	0.03
	Fabaceae	*Aeschynomene histrix*	0.15	Fabaceae	*Chamaecrista diphylla*	0.22	Fabaceae	*Chamaecrista diphylla*	0.03
Shrub	Verbenaceae	*Lippia microphylla*	0.35	Connaraceae	*Connarus favosus*	0.22	Trigoniaceae	*Trigonia villosa*	0.08
	Rubiaceae	*Palicourea rigida*	0.06	Trigoniaceae	*Trigonia villosa*	0.12	Verbenaceae	*Lippia microphylla*	0.03
	Connaraceae	*Rourea grosourdyana*	0.06	Verbenaceae	*Lippia microphylla*	0.1	-	-	-
Tree	Dilleniaceae	*Curatella americana*	2.72	Malpighiaceae	*Byrsonima crassifolia*	1.05	-	-	-
	Malpighiaceae	*Byrsonima crassifolia*	0.21	Erythroxylaceae	*Erythroxylum suberosum*	0.34	-	-	-
	Malpighiaceae	*Byrsonima coccolobifolia*	0.18	Proteaceae	*Roupala montana*	0.32	-	-	-
